# Primary Rectal Cancer: Repeatability of Global and Local-Regional MR Imaging Texture Features

**DOI:** 10.1148/radiol.2017161375

**Published:** 2017-05-05

**Authors:** Sofia Gourtsoyianni, Georgia Doumou, Davide Prezzi, Benjamin Taylor, J. James Stirling, N. Jane Taylor, Musib Siddique, Gary J. R. Cook, Robert Glynne-Jones, Vicky Goh

**Affiliations:** From the Department of Radiology (S.G., D.P., V.G.) and PET Centre (J.J.S., G.J.R.C.), Guy’s and St Thomas’ Hospitals NHS Foundation Trust, Level 1, Lambeth Wing, St Thomas’ Hospital, Westminster Bridge Road, London, SE1 7EH; Division of Imaging Sciences and Biomedical Engineering, King’s College London, London, England (G.D., D.P., B.T., J.J.S., M.S., G.J.R.C., V.G.); and the Cancer Centre, Mount Vernon Hospital, Northwood, England (N.J.T., R.G.).

## Abstract

**Purpose:**

To assess the day-to-day repeatability of global and local-regional magnetic resonance (MR) imaging texture features derived from primary rectal cancer.

**Materials and Methods:**

After ethical approval and patient informed consent were obtained, two pretreatment T2-weighted axial MR imaging studies performed prospectively with the same imaging unit on 2 consecutive days in 14 patients with rectal cancer (11 men [mean age, 61.7 years], three women [mean age, 70.0 years]) were analyzed to extract *(a)* global first-order statistical histogram and model-based fractal features reflecting the whole-tumor voxel intensity histogram distribution and repeating patterns, respectively, without spatial information and *(b)* local-regional second-order and high-order statistical texture features reflecting the intensity and spatial interrelationships between adjacent in-plane or multiplanar voxels or regions, respectively. Repeatability was assessed for 46 texture features, and mean difference, 95% limits of agreement, within-subject coefficient of variation (wCV), and repeatability coefficient *(r)* were recorded.

**Results:**

Repeatability was better for global parameters than for most local-regional parameters. In particular, histogram mean, median, and entropy, fractal dimension mean and standard deviation, and second-order entropy, homogeneity, difference entropy, and inverse difference moment demonstrated good repeatability, with narrow limits of agreement and wCVs of 10% or lower. Repeatability was poorest for the following high-order gray-level run-length (GLRL) gray-level zone size matrix (GLZSM) and neighborhood gray-tone difference matrix (NGTDM) parameters: GLRL intensity variability, GLZSM short-zone emphasis, GLZSM intensity nonuniformity, GLZSM intensity variability, GLZSM size zone variability, and NGTDM complexity, demonstrating wider agreement limits and wCVs of 50% or greater.

**Conclusion:**

MR imaging repeatability is better for global texture parameters than for local-regional texture parameters, indicating that global texture parameters should be sufficiently robust for clinical practice.

*Online supplemental material is available for this article.*

## Introduction

It is now recognized that tumors may demonstrate considerable biologic heterogeneity that influences their clinical outcome (1). Heterogeneity is also apparent phenotypically and can be appreciated in medical images (2) where traditionally radiology descriptors such as heterogeneous signal intensity or enhancement, mixed signal pattern, presence of areas of necrosis, hemorrhage, or calcification have been used to capture such morphologic information. Descriptive approaches have a clinical role but have limitations, and there has been increasing interest in alternative, quantitative method to portray this information, facilitated by the availability of texture analysis software platforms in clinical and research practice (3–6).

Results of a number of magnetic resonance (MR) imaging–based studies have now suggested that texture analysis may augment conventional imaging in cancer therapy response assessment (7–10) and may have a role as a prognostic and predictive biomarker, either alone or combined with clinical and genomic information (7,11). To date, there have been few studies assessing the robustness of such analysis (12–16). Image heterogeneity may be quantified by a number of postprocessing methods that address the variation of gray levels within a volume of interest, either on a global (whole-tumor) or local-regional (intratumoral, in-plane, or multiplanar) scale (Table 1, Tables E1–E7 [online]). These include *(a)* global model-based parameters such as fractal dimension and lacunarity, *(b)* global first-order statistical parameters derived from the gray-level intensity histograms (eg, mean, median, skewness, kurtosis, first-order entropy, and energy, also known as uniformity), *(c)* local second-order statistical parameters derived by using co-occurrence matrixes that reflect the spatial and signal intensity interrelationships between adjacent in-plane voxels (eg, contrast, homogeneity, second-order entropy, and energy), and *(d)* local-regional high-order parameters that examine the relationship between neighboring voxels in adjacent imaging planes (these include NGTDM parameters [eg, coarseness, contrast, busyness, complexity], which describe the dynamic range of intensities at a local level; run-length parameters, which describe the variation in signal intensity level across longitudinal voxels; and variations in zones or regions of intensity). The repeatability of these different texture parameters remains unknown in rectal cancer, there being no published studies with MR imaging to date (to our knowledge). Repeatability, an indication of measurement error, is essential information for clinical trials and future clinical practice; for example, if these quantitative parameters are used to augment therapy assessment. Thus, the aim of our study was to assess the day-to-day repeatability of global and local-regional MR imaging texture features derived from primary rectal cancer.

**Table 1:**
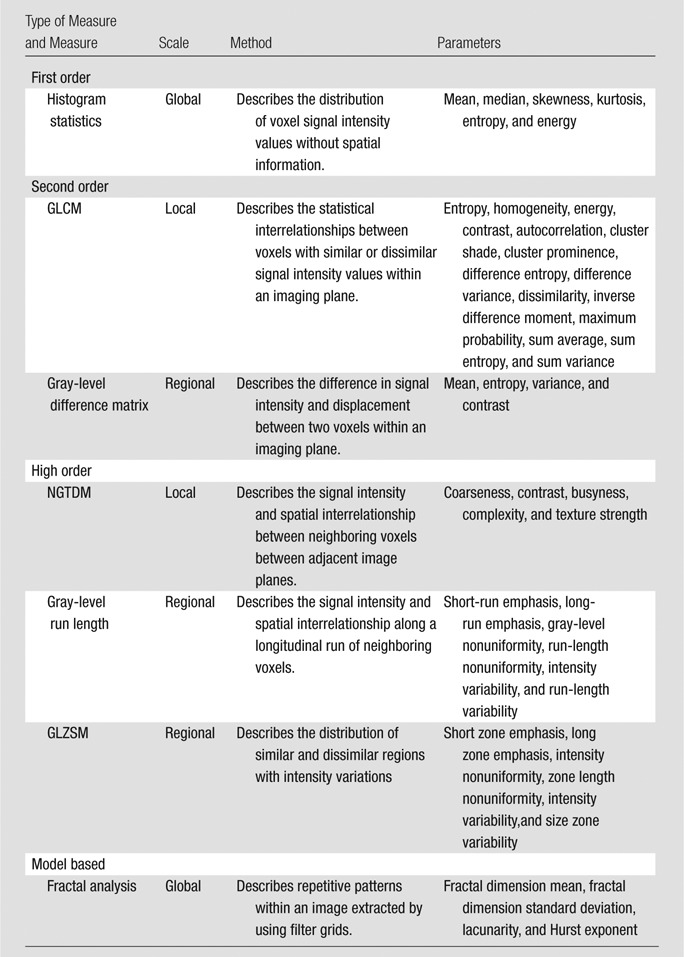
Summary of Global and Local-Regional Features Extracted for Each Tumor

Note.—GLCM = gray-level co-occurrence matrix, GLZSM = gray-level zone size matrix (Figure), NGTDM = neighborhood gray-tone difference matrix.

## Materials and Methods

### Patients

Institutional review board approval and informed consent were obtained for this study. Adults with locally advanced rectal cancer (stage ≥ T3N-positive or T4N0) who were scheduled to undergo chemotherapy and radiation therapy (45 Gy in 25 fractions with concomitant capecitabine 850 mg/m^2^) prior to surgery were recruited prospectively for a study investigating response biomarkers to preoperative chemotherapy and radiation therapy from May 2009 through January 2012. Exclusion criteria were contraindications to contrast material–enhanced MR imaging and metastatic disease at staging. Seventeen patients initially consented to undergo two pretreatment MR imaging examinations on consecutive days. Two patients subsequently declined a second baseline MR imaging examination, and one had hepatic metastatic disease, leaving a study group of 14 patients (11 men [mean age, 61.7 years; range, 52–79 years] and three women [mean age, 70.0 years; range, 60–78 years]).

### MR Imaging and Image Analysis

The pretreatment baseline MR imaging examinations were performed on consecutive days to allow assessment of day-to-day repeatability by using a 1.5-T system (Avanto and Symphony; Siemens, Erlangen, Germany). Both MR imaging examinations in each patient were performed on the same imaging unit by the same operator. No distention of the rectum was performed, and no rectal contrast material was administered. The T2-weighted axial turbo spin-echo pelvic MR images (repetition time msec/echo time msec, 4000/100; field of view, 263 × 350; matrix, 288 × 512 mm; section thickness, 6 mm; intersection gap, 1.8 mm) were selected and exported for further analysis. Texture analysis was performed by using an in-house program based on Matlab (Mathworks, Natick, Mass) to extract global whole-tumor and local-regional intratumoral features by using first-order, second-order, and high-order statistical methods and model-based methods (Table 1, Tables E1–E7 [online]). Image analysis was performed separately by two readers (G.D. [reader 1] and S.G. [reader 2], with 1 year and > 10 years of experience in MR imaging, respectively).

A region of interest was defined around the visualized tumor border by each reader on each axial image to segment a tumor volume for analysis (Figure), and subsequent analysis was automated within the software platform. A medium smoothing filter and 32-bin width were applied for quantization by the software. Global and local-regional texture features as listed in Table 1 and Tables E1–E7 (online) were extracted automatically for the defined tumor volume. This process was repeated for the second baseline MR imaging study, and the same global whole-tumor and local-regional intratumoral texture features were extracted for the defined tumor volume.

**Figure 1:**
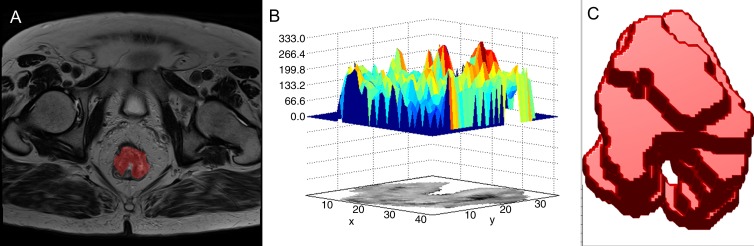
Segmentation of a rectal tumor for heterogeneity analysis: An example of tumor delineation on, *A,* a single axial MR image, *B,* a corresponding surface histogram plot, and, *C,* a subsequent whole-tumor composite volume on which analysis was performed.

### Statistical Analysis

To assess repeatability, the mean difference and 95% limits of agreement (with 95% confidence intervals [CIs] for the upper and lower limits of agreement) were determined for each of the texture parameters (17) for each observer. The mean difference reflects the difference between two separate individual measurements, while the limits of agreement provide the 95% CIs for the difference between individual measurements between different studies.

The within-subject coefficient of variation (wCV), representing the within-subject variability of parameters and expressed as a percentage, was calculated by dividing the within-subject standard deviation by the group mean as follows:
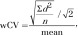
where *d* is the difference and *n* is the number of subjects. wCVs of greater than 50% were considered to indicate unreliability for clinical implementation.

The repeatability coefficient representing the precision of repeated measures was calculated as follows:
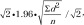
where *d* is the difference and *n* is the number of subjects. This is a 95% CI: The difference between two measurements for the same subject will be less than this value in 95% of cases.

## Results

There were 10 T3N1 tumors, three T3N2 tumors, and one T4N0 tumor; mean tumor length was 50 mm (range, 30–85 mm). The mean differences, 95% limits of agreement, wCVs, and repeatability coefficients for the 46 image texture parameters are summarized in Tables 2–8 for both observers. Day-to-day repeatability was variable depending on the class and type of parameter.

**Table 2:**
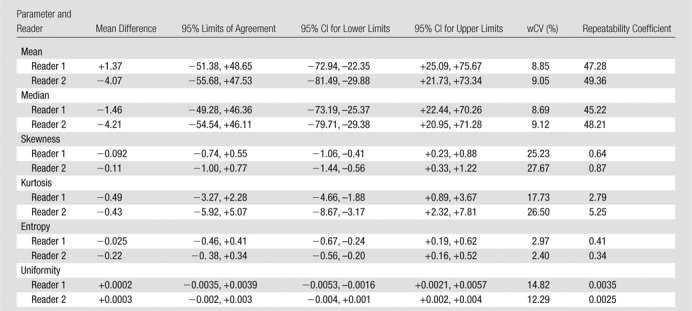
Repeatability between Two Baseline MR Imaging Examinations for Global First-Order Statistical Histogram Parameters

**Table 3:**
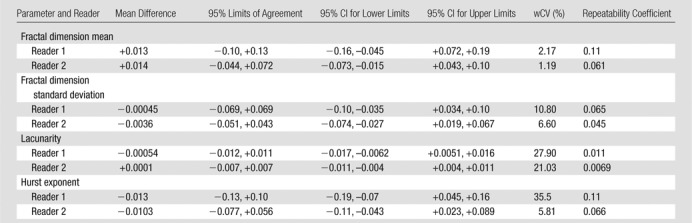
Repeatability between Two Baseline MR Imaging Examinations for Global Fractal Parameters

**Table 4:**
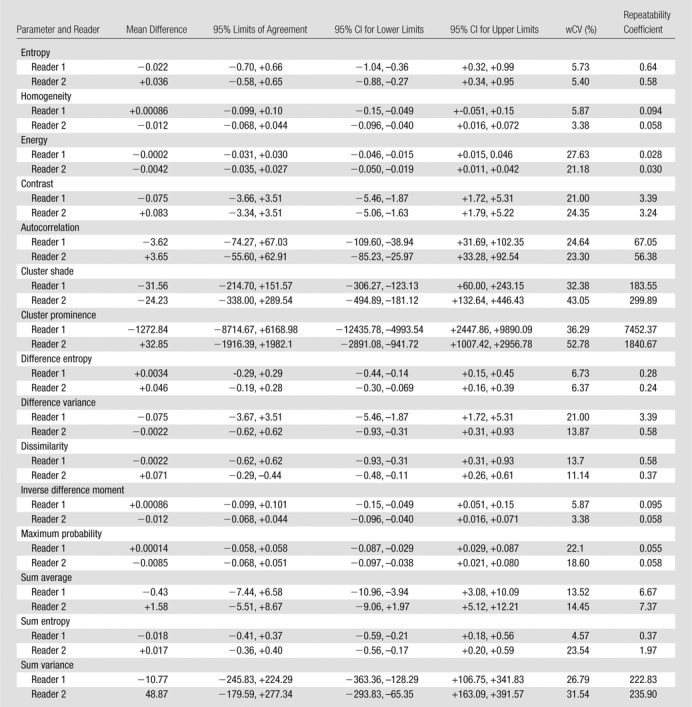
Repeatability between Two Baseline MR Imaging Examinations for Local-Regional Second-Order Gray-Level Co-occurrence Matrix Texture Parameters

**Table 5:**
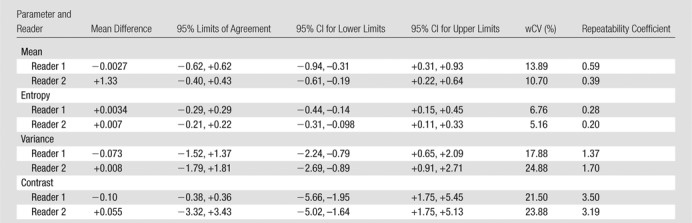
Repeatability between Two Baseline MR Imaging Examinations for Local-Regional Second-Order Gray-Level Difference Matrix Texture Parameters

**Table 6:**
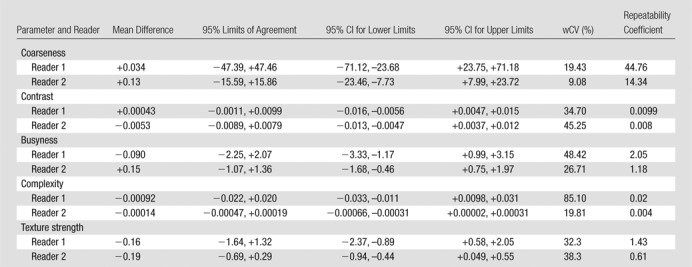
Repeatability between Two Baseline MR Imaging Examinations for Local-Regional High-Order Neighborhood Gray-Tone Difference Matrix Texture Parameters

**Table 7:**
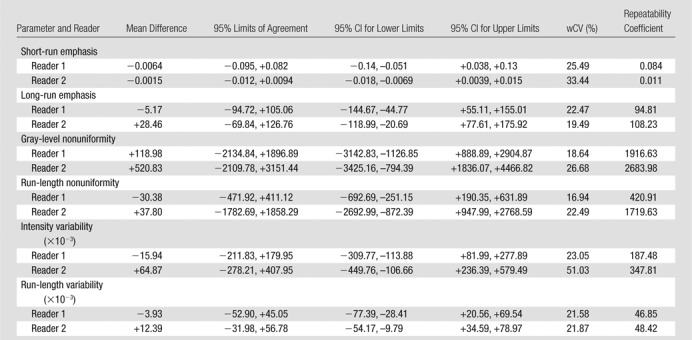
Repeatability between Two Baseline MR Imaging Examinations for Local-Regional High-Order Gray-Level Run-Length Texture Parameters

**Table 8:**
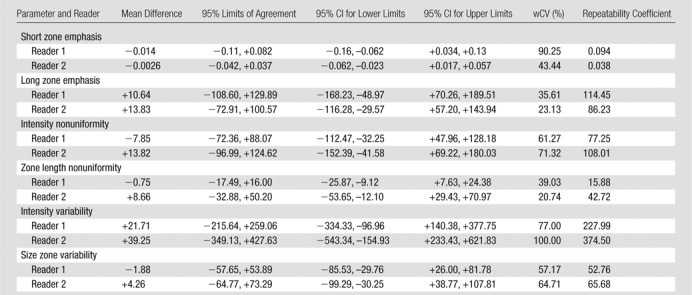
Repeatability between Two Baseline MR Imaging Examinations for Local-Regional High-Order GLZSMgray-level zone size matrix Texture Parameters

Repeatability was generally better for whole-tumor global parameters (Tables 2, 3) than for intratumoral local-regional parameters, with the exception of first-order skewness, kurtosis, fractal lacunarity, and Hurst exponent. In particular, first-order mean, median, and entropy and fractal dimension mean and standard deviation demonstrated narrow limits of agreement, with low within-subject variability of 10% or less. The repeatability of first-order energy was also acceptable, with the within-subject variability ranging from 10% to 15%.

For second-order in-plane local-regional parameters (Tables 4, 5), only GLCM entropy, GLCM homogeneity, GLCM difference entropy, and GLCM inverse difference moment demonstrated narrow limits of agreement with low within-subject variability of 10% or less. Cluster shade, cluster prominence, and sum entropy demonstrated only moderate limits of agreement and within-subject variability of 30% or more for at least one observer.

Repeatability was poorest for high-order local-regional parameters (Tables 6–8), which reflects the interrelationship between multiplanar voxels. GLZSM and NGTDM parameters, with the exception of NGTDM coarseness, demonstrated poorer limits of agreement and within-subject variability of 30% or greater for at least one observer. In particular, NGTDM complexity, gray-level run-length intensity variability, GLZSM short-zone emphasis, GLZSM intensity nonuniformity, GLZSM intensity variability, and GLZSM size zone variability demonstrated within-subject variability of 50% or greater.

## Discussion

Low measurement error is essential for any quantitative technique to be translated into clinical practice. In our study, we found that global methods that reflect the voxel distribution within the whole tumor demonstrated higher repeatability than did local-regional methods that describe the intervoxel relationships on a local or regional level. Specifically, global first-order mean, median, and entropy and fractal dimension mean and standard deviation demonstrated narrow limits of agreement, with low within-subject variability of 10% or less. First-order energy was also acceptable, with the within-subject variability ranging from 10% to 15% and thus being sufficiently robust for clinical practice. Of the local-regional parameters, gray-level difference matrix entropy, GLCM entropy, GLCM homogeneity, GLCM difference entropy, and GLCM inverse difference moment demonstrated the narrowest limits of agreement, from −0.29 to +0.29, −0.70 to +0.66, −0.099 to +0.10, −0.29 to +0.29, and −0.099 to +0.101, respectively.

Repeatability was poorest for high-order local-regional parameters. GLZSM and NGTDM parameters, with the exception of NGTDM coarseness, were poorly reproducible across the two MR imaging examinations. Although our observers had different levels of MR imaging experience, the level of repeatability was consistent for most parameters. Discrepancies, where present, were primarily related to high-order local-regional parameters—for example, NGTDM complexity, busyness, and contrast; gray-level run-length intensity and gray-level variability; and GLZSM short-zone emphasis. This is most likely to be related to the differences in voxels included in the volume of interest delineation by the observers.

There have been few studies investigating the repeatability of both global and local-regional texture parameters, and none have been published for MR imaging, to our knowledge. Fried et al (12) reported an average correlation coefficient of 0.67 for contrast-enhanced computed tomography (CT) of stage III non–small cell lung cancer (NSCLC) in 13 patients, with only 15 (23%) of the 66 features studied (first-order [histogram], second-order [GLCM] and high-order [NGTDM] statistical parameters, as well as Laplacian of Gaussian filtration metrics) showing a correlation coefficient greater than 0.9 (12). Similarly, Balagurunathan et al (13) reported that 66 (30.9%) texture features had a correlation coefficient greater than 0.9 in 32 patients with NSCLC undergoing noncontrast CT. Sanghera et al (14) found that fractal dimension and lacunarity features in perfusion CT blood flow maps demonstrated coefficients of variation of less than 7.35% between studies. A further study of fluorine 18 fluorodeoxyglucose positron emission tomography statistic-based textural features in 16 patients with esophageal cancer showed variable reliability, with intraclass correlation coefficients ranging from 0.59 to 0.99 (15). Our findings for T2-weighted MR imaging demonstrate a similar pattern and degree of repeatability.

The use of more than one statistical method to evaluate repeatability reflects the fact that they provide complementary information regarding interstudy agreement. In our study, we selected Bland-Altman 95% limits of agreement, wCVs, and the repeatability coefficient, as they provide a measure of the variability of the parameters itself, a measure of the variability for individuals within the study group, and the degree of measured parameter change that is likely to be beyond day-to-day natural measurement variation in the therapy response setting.

Assessment of the repeatability of MR imaging texture parameters is highly relevant to rectal cancer. MR imaging plays a key role in the treatment of these patients (18). The additional information from texture analysis (3) may contribute to a more personalized management approach by providing prognostic and predictive information. Global first-order statistic-based (mean, standard deviation, skewness, kurtosis, entropy, and energy) and model-based metrics (fractal dimension, lacunarity) have been used most commonly to date because of their availability through commercial and open-access research software platforms. Initial studies have suggested that global and local-regional features extracted from the primary tumor may predict for nodal metastases (19) and overall survival at CT (20), as well as treatment response at MR imaging (7). De Cecco et al (7) found that T2-weighted filtered global statistical texture parameters differed between responders and nonresponders in an exploratory study of 15 patients with rectal cancer; baseline kurtosis with a medium texture scale was lower in the complete responder group. Complete responders also demonstrated greater changes in kurtosis in the middle of treatment than did nonresponders. A further retrospective exploratory dynamic contrast-enhanced MR imaging–based study of 10 patients with 26 colorectal cancer liver metastases treated with bevacizumab and oxaliplatin–5 fluorouracil–leucovorin that used global statistical and fractal method texture analysis showed that pretreatment low-box dimension is associated with a better tumor response (9).

Nevertheless, there were limitations to our study. We chose to analyze T2-weighted images acquired by using a turbo spin-echo sequence specifically because these sequences are performed universally in clinical practice and have been shown to provide predictive and prognostic information in rectal cancer (18,21,22); however, our findings may not be generalizable to other MR imaging sequences that may be performed to assess rectal cancer (including T1-weighted, diffusion-weighted, and dynamic contrast-enhanced MR imaging). It has been shown that texture analysis can be affected by the MR imaging acquisition and postprocessing methods (23–25); spatial resolution may be the most important factor for MR imaging (23,24). However, one group of researchers (24) has suggested that if the spatial resolution is sufficiently high, variations in repetition time, echo time, and number of averages have little effect; indeed, texture features derived from the co-occurrence matrix were the most robust in their study (23). Other groups have suggested that signal-to-noise ratio, field strength (1.5 vs 3.0 T), and intensity normalization algorithms (25) influence texture analysis, highlighting the importance of consistent methods. In our study, the MR imaging sequences were performed as part of a prospective research protocol and were therefore subject to standardization and quality control. Indeed, the same MR imaging unit and operator were used for each patient. Therefore, our results reflect a “best-case scenario” that may not necessarily be directly translatable to different MR imaging units with different protocols. However, the knowledge of the degree of variation in general remains relevant to future clinical practice. Our patient numbers were also small, reflecting the challenges of obtaining consent for a second acquisition that will not alter treatment and that is not perceived as having a direct clinical benefit.

In conclusion, few studies to date have assessed the repeatability of image texture parameters. We found that the day-to-day fluctuations in MR imaging texture parameters were lower for global parameters than for local-regional parameters. In particular, first-order histogram (mean, median, entropy) and model-based fractal parameters (fractal dimension mean, fractal dimension standard deviation) consistently demonstrated good repeatability, with narrow limits of agreement and a wCV of less than 10%, which is acceptable for clinical practice.

**Advances in Knowledge**

■ Several texture parameters demonstrated good repeatability, with narrow limits of agreement and within-subject coefficients of variation (wCVs) of 10% or lower.
■ Global parameters that provided a global measure of signal distribution and demonstrated good repeatability included *(a)* histogram parameters (first-order histogram mean, median, and entropy) and *(b)* model-based fractal dimension mean and standard deviation.
■ Local parameters within a defined section within the lesion that reflected the intratumoral intensity and spatial interrelationships between adjacent in-plane voxels and demonstrated good repeatability included second-order gray-level co-occurrence matrix entropy, homogeneity, difference entropy, and inverse difference moment.
■ Repeatability was poorest for high-order texture parameters that provided information about intratumoral local-regional intensity and spatial interrelationships between multiplanar voxels or regions (in particular, several neighborhood gray-tone difference matrix and gray-level zone size parameters showed wCVs of 50% or greater).

**Implication for Patient Care**

■ The day-to-day repeatability of MR imaging global whole-tumor texture parameters is excellent for rectal cancer and opens the possibility of future clinical usage of whole-tumor MR imaging texture analysis in view of its potential prognostic information.

## Supplemental Material

Tables E1–E7 (PDF)
