# Supplementary material for: Primary Rectal Cancer: Repeatability of Global and Local-Regional MR Imaging Texture Features
Source: Radiology. 2017 May 5;284(2):552–61. doi: 10.1148/radiol.2017161375 (PMC6150741; doi:10.1148/radiol.2017161375)
Supplement: Tables E1–E7 (PDF) [file ry161375suppa1.pdf]

©RSNA, 2017
10.1148/radiol.2017161375

Table E1. Summary Description and Relevant Formulas for First-Order Histogram Statistics

Parameter	Description	Formula
Mean	Describes the average of all voxel signal intensities.	Let *N* be the number of voxels in a three-dimensional region of interest and *I(i)* the gray level at a particular voxel *i*. $$\mu = \frac{1}{N}\sum_{i=1}^{N} I(i)$$
Median	Describes the signal intensity value in the middle when voxels are ranked in ascending order	…
Skewness	Measures signal asymmetry and deviation from a normal distribution. Skewness > 0: right skewed, values concentrated on the left of the mean. Skewness < 0: left skewed, values concentrated on the right of the mean. Skewness = 0: normal distribution.	$$s = \frac{1}{N}\sum_{i=1}^{N}\left[\frac{I(i)-\mu}{\sigma}\right]^3$$ where $$\sigma = \sqrt{\frac{1}{N}\sum_{i=1}^{N}[I(i)-\mu]^2}$$
Kurtosis	Describes the "peak" of a distribution. Kurtosis > 3: sharper peak than a normal distribution. Kurtosis < 3: flatter peak than a normal distribution. Kurtosis = 3: normal distribution.	$$k = \frac{1}{\mathrm{N}}\sum_{i=1}^{N}\left[\frac{I(i)-\mu}{\sigma}\right]^4$$
Energy	Measures voxel signal distribution. High energy is noted in homogeneous voxels	$$\text{energy} = \sum_{i=1}^{N_g}[p(i)]^2$$ Where p is a vector that contains the histogram counts and N_g is total number of different gray levels present in the image.
Entropy	Measures voxel randomness. Low entropy is noted in homogeneous voxels.	$$\text{entropy} = -\sum_{i=1}^{N_g} p(i)\log_2 p(i)$$ Where p is a vector that contains the histogram counts and N_g is total number of different gray levels present in the image.

Note.—These statistics provide an indication of central tendency (mean, median) and variability (skewness, kurtosis, energy, and entropy).

Table E2. Summary Description and Relevant Formulas for Second-Order GLCM Statistics

Parameter	Description	Formula

Entropy$_{GLCM}$	Measures the local randomness within the image. Homogeneous textures have low entropy$_{GLCM}$.	$-\sum_i \sum_j p(i,j) \ln\left[p(i,j)\right]$,<br>Where *i* is the voxel value between *i* = 1 and i_{max} in the region of interest, *j* is the voxel value between *j* = 1 and j_{max} in the region of interest, and *p(i,j)* is the probability of the occurrence of that voxel value *i* relative to *j*.
Homogeneity	Measures the relative homogeneity between voxels. The value is high for homogeneous images.	$\sum_i \sum_j \frac{1}{1+(i+j)^2} p(i,j)$,<br>Where *p(i,j)* is the probability of the occurrence of that voxel value *i* relative to *j*.
Energy$_{GLCM}$	Measures homogeneity. Homogeneous images demonstrate high energy.	$\sum_i \sum_j \{p(i,j)\}^2$,<br>*p(i,j)* is the probability of the occurrence of that voxel value *i* relative to *j*.
Contrast$_{GLCM}$	Measures local gray-level intensity variation and favors contributions from voxels *p(i, j)* away from the diagonal of p (ie, i ≠ j).	$\sum_{n=0}^{N_g-1} n^2 \left\{ \sum_{i=1}^{N_g} \sum_{j=1}^{N_g} p(i,j) \middle\| \|i-j\| = n \right\}$,<br>Where *i* is the voxel value between *i* = 1 and i_{max} in the region of interest, *j* is the voxel value between *j* = 1 and j_{max} in the region of interest, *n* is the number of voxels, and *p(i,j)* is the probability of the occurrence of that voxel value *i* relative to *j*. N_g is the number of distinct gray levels in the quantized image.
Autocorrelation	Measures gray-level intensity linear dependence between the voxels *(i,j)* at the specified positions relative to each other.	$\sum_i \sum_j (ij)\, p(i,j)$,<br>Where *i* is the voxel value between *i* = 1 and i_{max} in the region of interest, *j* is the voxel value between *j* = 1 and j_{max} in the region of interest, and *p(i,j)* is the probability of the occurrence of that voxel value *i* relative to *j*.
Cluster shade	Measures the skewness of the matrix and is thought to gauge the perceptual concepts of uniformity. When the cluster shade is high, the image is more skewed.	$\sum_i \sum_j (i+j-\mu_x-\mu_y)^3 p(i,j)$,<br>Where *i* is the voxel value between *i* = 1 and i_{max} in the region of interest, *j* is the voxel value between *j* = 1 and j_{max} in the region of interest, and *p(i,j)* is the probability of the occurrence of that voxel value *i* relative to *j*. μ_x Is the mean of p_x, whereas μ_y is the mean of p_y. p_x Is obtained by summing rows of p that is, $\sum_j^{N_g} p(i,j)$, and p_y is obtained by summing columns of p that is, $\sum_i^{N_g} p(i,j)$. N_g is the number of distinct gray levels in the quantized image.
Cluster prominence	Measures asymmetry. A low cluster prominence value indicates small variations in grayscale.	$\sum_i \sum_j (i+j-\mu_x-\mu_y)^4 p(i,j)$,<br>Where *i* is the voxel value between *i* = 1 and i_{max} in the region of interest, *j* is the voxel value between *j* = 1 and j_{max} in the region of interest, *p(i,j)* is the probability of the occurrence of that voxel value *i* relative to *j*, μ_x is the mean of p_x, and μ_y is the mean of p_y. p_x Is obtained by

		summing rows of p that is, $\sum_{j}^{N_g} p(i,j)$ and p_y is obtained by summing columns of p that is, $\sum_{i}^{N_g} p(i,j)$.
Difference entropy	Measure of the inhomogeneity of the image. High difference entropy reflects greater inhomogeneity.	$-\sum_{i=0}^{N_g-1} p_{x-y}(i)\log\left[p_{x-y}(i)\right]$, Where N_g is the number of quantized gray levels in image G, i is the voxel value between $i = 1$ and i_{max} in the region of interest, and $p_{(x-y)}$ is the probability of the occurrence of that voxel value x relative to y.
Difference variance	Measure of the inhomogeneity of the image. High difference variance reflects greater inhomogeneity.	$\sum_{i=0}^{N_g-1} \left(i-\mu_{x-y}\right)^2 p_{x-y}(i)$, Where N_g is the number of quantized gray levels in image G, i is the voxel value between $i = 1$ and i_{max} in the region of interest, $p_{(x-y)}$ is the probability of the occurrence of that voxel value x relative to y, and μ_{x-y} is the mean of p_{x-y}.
Dissimilarity	Measure of the inhomogeneity of the image. Dissimilarity is a high value for inhomogeneous images and a relatively lower value for homogeneous images.	$\sum_i \sum_j \lvert i-j\rvert^2 \; p(i,j)$, Where i is the voxel value between $i = 1$ and i_{max} in the region of interest, j is the voxel value between $j = 1$ and j_{max} in the region of interest, and $p(i,j)$ is the probability of the occurrence of that voxel value i relative to j.
Inverse difference moment	Measures the relative homogeneity between voxels.	$\sum_i \sum_j \frac{1}{1+\lvert i-j\rvert} p(i,j)$, Where i is the voxel value between $i = 1$ and i_{max} in the region of interest, j is the voxel value between $j = 1$ and j_{max} in the region of interest, and $p(i,j)$ is the probability of the occurrence of that voxel value i relative to j.
Maximum probability	Measures the maximum probability of pixel combination. High values occur if one combination of pixels dominates the pixel pairs in the image.	$\max p(i,j)$ Where $p(i,j)$ is the probability of the occurrence of that voxel value i relative to j.
Sum average	High-contrast regions have high values, while low-contrast regions will have low values.	$\sum_{i=2}^{2N_g} i \, p_{x+y}(i)$, Where $p_{x+y}(i) = \sum_x \sum_y p(x,y)$ where $x+y=i$ and $i = 2,3,\ldots,2N_g$
Sum entropy	Measure of the inhomogeneity of the image. High sum entropy reflects greater inhomogeneity.	$-\sum_{n=2}^{2N_g} p_{x+y}(n)\log\left[p_{x+y}(n)\right]$ Where N_g is the number of quantized gray levels in image G and

		$p_{x+y}(n) = \sum_x \sum_y p(x,y)$ where $x + y = n$ and $n = 2,3,\ldots,2N_g$
Sum variance	Measure of the inhomogeneity of the image. High sum variance reflects greater inhomogeneity.	$\sum_{i=2}^{2N_g} (i - \mu_{x+y})^2 \, p_{x+y}(i)$, Where N_g is the number of quantized gray levels in image G, i is the voxel value between $i = 1$ and i_{max} in the region of interest. $p_{x+y}(i) = \sum_x \sum_y p(x,y)$ where $x + y = i$ and $i = 2,3,\ldots,2N_g$

Note.—An original texture image, D, is requantized into an image G with a reduced number of gray levels, N_g. A typical value of N_g is 16 or 32. Then, GLCM is computed from G by scanning the intensity of each voxel and its neighbor, defined by displacement d and angle θ. Displacement d could take a value of 1,2,3,…n, whereas angle θ was limited to 0°, 45°, 90° and 135°. The GLCM $p(i, j \mid d, \theta)$ is a second-order joint probability density function of gray-level pairs in the image for each element in the co-occurrence matrix obtained by dividing each element with N_g. Finally, scalar secondary features are extracted from this co-occurrence matrix.

Table E3: Summary Description and Relevant Formulas for Second-Order Gray-Level Difference Matrix Statistics

Parameter	Description	Formula
Mean_{GLDM}	Measures the mean gray-level difference. Small μ_d values indicate a coarse texture equal to or larger than the magnitude of the displacement vector.	$\mu_d = \sum_{k=1}^{N} g_k p_g(g_k, d)$, Where N is the number of gray levels, g_k is the gray level within the voxel, p_g is the histogram of the gray-level difference at specific distance d, and d is the displacement vector.
Entropy_{GLDM}	Measures the homogeneity of the histogram. It is maximized for uniform histograms.	$H_d = \sum_{k=1}^{N} p_g(g_k, d) \ln p_g(g_k, d)$, Where N is the number of gray levels, g_k is the gray level within the voxel, p_g is the histogram of the gray-level difference at specific distance d, and d is the displacement vector.
Variance	Measures the dispersion of gray-level differences at a certain distance, d.	$\sigma_d^2 = \sum_{k=1}^{N} (g_k - \mu_d)^2 \, p_g(g_k, d)$, Where μ_d is the mean difference, N is the number of gray levels, g_k is the gray level within the voxel, p_g is the histogram of the gray-level difference at specific distance d, and d is the displacement vector.
Contrast_{GLDM}	Measures the local variations in gray-level difference in the image.	$C_d = \sum_{k=1}^{N} g_k^2 p_g(g_k, d)$, Where N is the number of gray levels, g_k is the gray level within the voxel, p_g is the histogram of the gray-level difference at specific distance d, and d is the displacement vector.

Note.—GLDM = gray-level difference matrix. The gray-level differences are computed by taking the absolute differences of all possible pairs of gray levels distance d apart at angle Θ and counting the number of times the difference is 0,1,…, 255. d Is (dx,dy), the displacement vector between two image pixels, and

$g(d)$ is the gray-level difference at distance d. $p_g(g,d)$ Is the histogram of the gray-level differences at the specific distance d. One distinct histogram exists for each distance d. The difference statistics are then normalized by dividing each element of the vector by the number of possible pixel pairs. Several texture measures can be extracted from the histogram of gray-level differences. N is the number of gray levels.

Table E4. Summary Description and Relevant Formulas for High-Order NGTDM Statistics

Parameter	Description	Formula
Coarseness	Measures gray-tone difference within the image. High coarseness represents areas where gray-tone differences are small. Small gray-tone differences possess a high degree of local uniformity in intensity.	$f_{\cos} = \left[\epsilon + \sum_{i=0}^{G_h} p_i s(i)\right]^{-1}$ and $p_i = N_i / n^2$, Where s is the NGTDM vector, p_i is the probability of a voxel value for voxels, n is the number of unique gray levels present in an image, and G_h is the highest gray level in an image.
Contrast$_{NGTM}$	Measures local variation in intensity. High contrast indicates areas of large intensity difference between neighboring regions.	$\left[\frac{1}{N_g(N_g-1)}\sum_{i=0}^{G_h}\sum_{j=0}^{G_h} p_i p_j (i-j)^2\right]\left[\frac{1}{n^2}\sum_{i=0}^{G_h} s(i)\right]$ and $N_g = \sum_{i=0}^{G_h} Q_i$, where $Q_i = \begin{cases}1\\0\end{cases}$,if $p_i \neq 0$ otherwise , Where s is the NGTDM vector; p_i is the probability of a voxel value for voxels, n is the number of unique gray levels present in an image, and G_h is the highest gray level in an image.
Busyness	Measures changes in intensity from one voxel to its neighbor; high busyness indicates that the spatial frequency of intensity changes is very high.	$\left[\sum_{i=0}^{G_h} p_i s(i)\right] / \left[\sum_{i=0}^{G_h}\sum_{j=0}^{G_h} ip_i - jp_j\right]$ and $(p_i \neq 0 , p_j \neq 0)$, Where s is the NGTDM vector, p_i is the probability of a voxel value for voxels, n is the number of unique gray levels present in an image, and G_h is the highest gray level in an image.
Complexity	Measures the texture information content. High values of complexity indicate a high degree of information content where there are many voxels with different average intensities.	$\sum_{i=0}^{G_h}\sum_{=0}^{G_h}\left\{(\lvert i-j\rvert) / \left[n^2 (p_i + p_j)\right]\right\}\left\{p_i s(i) + p_j s(j)\right\}$ and $(p_i \neq 0, p_j \neq 0)$, Where s is the NGTDM vector, p_i is the probability of a voxel value for voxels, n is the number of unique gray levels present in an image, and G_h is the highest gray level in an image.
Texture strength	Measures the strength of signal intensity. A texture is generally referred to as strong when voxels are easily definable and clearly visible.	$\left[\sum_{i=0}^{G_h}\sum_{j=0}^{G_h} (p_i + p_j)(i+j)^2\right] \big/ \left[\epsilon + \sum_{i=0}^{G_h} s(i)\right]$ and $(p_i \neq 0, p_j \neq 0)$, Where s is the NGTDM vector, p_i is the probability of a voxel value for voxels, n is the number of unique gray levels present in an image, and G_h is the highest gray level in an image.

Note.—NGTDM statistics describe the local intensity differences between each voxel and its direct 26 neighbors.

Table E5. Summary Description and Relevant Formulas for High-Order Gray-Level Run-Length Statistics

Parameter	Description	Formula
Short run emphasis	Measures the occurrence and distribution of short runs. Higher values are expected for fine textures.	$\frac{1}{n_r}\sum_{i=1}^{M}\sum_{j=1}^{N}\frac{p(i,j)}{j^2}$, Where *i* is the gray-level intensity, *j* is the number of runs, *p(i,j)* is the GLRLM entry at displacement *d* and displacement *d(x, y, z)*, *M* is the number of rows of *p* and represents the maximum gray level in the image, *N* is the number of columns of *p* and represents the maximum run length in the image, and n_r is the total number of runs in the region of interest.
Long run emphasis	Measures the occurrence and distribution of long runs. Higher values expected for coarse textures.	$\frac{1}{n_r}\sum_{i=1}^{M}\sum_{j=1}^{N}p(i,j)\cdot j^2$, Where *i* is the gray-level intensity, *j* is the number of runs, *p(i,j)* is the GLRLM entry at displacement *d* and displacement *d(x, y, z)*, *M* is the number of rows of *p* and represents the maximum gray level in the image, *N* is the number of columns of *p* and represents the maximum run length in the image, and n_r is the total number of runs in the region of interest.
Gray-level nonuniformity	Measures the similarity of gray-level values throughout the image. Smaller values are expected for similar gray-level values in an image.	$\frac{1}{n_r}\sum_{i=1}^{M}\left(\sum_{j=1}^{N}P(i,j)\right)^2$, Where *i* is the gray-level intensity, *j* is the number of runs, *p(i,j)* is the GLRLM entry at displacement *d* and displacement *d(x, y, z)*, *M* is the number of rows of *p* and represents the maximum gray level in the image, *N* is the number of columns of *p* and represents the maximum run length in the image, and n_r is the total number of runs in the region of interest.
Run-length nonuniformity	Measures the similarity of the length of runs throughout the image. The run-length nonuniformity is expected to be small if the run lengths are alike throughout the image.	$\frac{1}{n_r}\sum_{j=1}^{N}\left(\sum_{i=1}^{M}p(i,j)\right)^2$, Where *i* is the gray-level intensity, *j* is the number of runs, *p(i,j)* is the GLRLM entry at displacement *d* and displacement *d(x, y, z)*, *M* is the number of rows of *p* and represents the maximum gray level in the image, *N* is the number of columns of *p* and represents the maximum run length in the image, and n_r is the total number of runs in the region of interest.
Intensity variability	Measures the similarity of gray-level values throughout the image. It is small if the gray-level values are alike throughout the image.	$\frac{1}{n_r}\sum_{i=1}^{M}\left[\sum_{j=1}^{N}\frac{p(i,j)}{i^2}\right]^2$, Where *i* is the gray-level intensity, *j* is the number of runs, *p(i,j)* is the GLRLM entry at displacement *d* and displacement *d(x, y, z)*, *M* is the number of rows of *p* and represents the maximum gray level in the image, *N* is the number of columns of *p* and represents the maximum run length in the image, and n_r is the total number of runs in the region of interest.

Run-length variability	Measures the similarity of the size of zones throughout the image. It is small if the run lengths are alike throughout the image.	$\frac{1}{n_r}\sum_{i=1}^{M}\left[\sum_{j=1}^{N}\frac{p(i,j)}{j^2}\right]^2$, Where i is the gray-level intensity, j is the number of runs, $p(i,j)$ is the GLRLM entry at displacement d and displacement $d(x, y, z)$, M is the number of rows of p and represents the maximum gray level in the image, N is the number of columns of p and represents the maximum run length in the image, and n_r is the total number of runs in the region of interest.

Note.—GLRLM = gray-level run-length matrix. GLRLMs calculate the number of texels, which describes the coarseness of the region or image. Texels or run lengths are pixels with the same intensity. Fine textures contain more short runs with similar gray-level intensities, while coarse textures have more long runs with significantly different gray-level intensities.

Table E6: Description and Formulas for Texture Feature Parameters Derived from GLSZM Statistics

Parameters	Description	Formula
Short-zone emphasis	Measures the distribution of small zones. It depends on the occurrence of small zones and is large for fine textures.	$\frac{1}{\Omega}\sum_{i=1}^{M}\sum_{j=1}^{N}\frac{z(i,j)}{j^2}$, Where $z(i,j)$ is the zone size matrix, with rows I indicating gray levels and columns j indicating zone sizes; M is the highest gray level; N is the largest zone size, and Ω is the total number of unique connected zones.
Long-zone emphasis	Measures the distribution of long runs. It depends on the occurrence of large zones and is large for coarse textures.	$\frac{1}{\Omega}\sum_{i=1}^{M}\sum_{j=1}^{N}j^2, z(i,j)$, Where $z(i,j)$ is the zone size matrix, with rows I indicating gray levels and columns j indicating zone sizes; M is the highest gray level; N is the largest zone size, and Ω is the total number of unique connected zones.
Intensity nonuniformity	Measures the similarity of gray-level values throughout the image. It is small if the gray-level values are alike throughout the image.	$\frac{1}{\Omega}\sum_{i=1}^{M}\left[\sum_{j=1}^{N}\frac{z(i,j)}{i^2}\right]^2$, Where $z(i,j)$ is the zone size matrix, with rows I indicating gray levels and columns j indicating zone sizes; M is the highest gray level; N is the largest zone size, and Ω is the total number of unique connected zones.
Zone percentage	Measures the homogeneity and distribution of zones of an image in a specific direction. It is the largest when the size of the zones is 1 for all gray levels.	$\frac{\Omega}{\sum_{i=1}^{M}\sum_{j=1}^{N}j^2}, z(i,j)$, Where $z(i,j)$ is the zone size matrix, with rows I indicating gray levels and columns j indicating zone sizes; M is the highest gray level; N is the largest zone size, and Ω is the total number of unique connected zones.

Intensity variability	Measures the similarity of gray-level values throughout the image. It is small if the gray-level values are alike throughout the image.	$$\frac{1}{\Omega}\sum_{i=1}^{M}\left[\sum_{j=1}^{N}\frac{z(i,j)}{i^2}\right]^2,$$ Where $z(i,j)$ is the zone size matrix, with rows I indicating gray levels and columns j indicating zone sizes; M is the highest gray level; N is the largest zone size, and Ω is the total number of unique connected zones.
Size zone variability	Measures the similarity of the size of zones throughout the image. It is small if the zone sizes are alike throughout the image.	$$\frac{1}{\Omega}\sum_{i=1}^{N}\left[\sum_{j=1}^{M}\frac{z(i,j)}{j^2}\right]^2,$$ Where $z(i,j)$ is the zone size matrix, with rows I indicating gray levels and columns j indicating zone sizes; M is the highest gray level; N is the largest zone size, and Ω is the total number of unique connected zones.

Note.—For a given three-dimensional image, a GLSZM Z is defined as follows: Each element $z(i,j)$ represents the number of zones with pixels of gray-level intensity equal to i and size of zone equal to j. The size of the matrix Z is $M \times N$, where M is the maximum gray level in the scan and N is equal to the possible maximum zones in the corresponding scan.

Table E7. Description and Formulas for Texture Feature Parameters Derived from Fractal Analysis

Parameters	Description	Formula
Mean fractal dimension	Measures the texture of a fractal, a self-similar pattern. A higher fractal dimension corresponds to greater roughness.	$$\bar{D} = \frac{\sum_{i=1}^{N} D_i}{N},$$ Where N is the number of sections and D_i is the fractal dimension for the ith section.
Standard deviation	Measures the standard deviation of a fractal computed by a differential box-counting algorithm.	$$\sigma = \sqrt{\sum_{i=1}^{N} D_i^2 / N - \left(\frac{\sum_{i=1}^{N} D_i}{N}\right)^2},$$ Where N is the number of sections and D_i is the fractal dimension for the ith section.
Lacunarity	Measures the amount of “gaps” in the image or object. If a fractal has large gaps, it has high lacunarity.	$$\frac{\left[\sum_{i=1}^{N} D_i^2 / N\right]}{\left[\left(\frac{\sum_{i=1}^{N} D_i}{N}\right)^2\right]} - 1,$$ Where N is the number of sections and D_i is the fractal dimension for the ith section.
Hurst component	Measures the density of the image or object (ie, how much the image or object occupies the space that contains it). A small value corresponds to a coarse texture.	$$H = 3 - \bar{D},$$ Where $\bar{D}$ is the mean fractal dimension.

Note.—Fractal features describe self-similar fractal shapes.
